# Information systems project success surveys - Insights from the last 30 years

**DOI:** 10.1016/j.heliyon.2024.e40619

**Published:** 2024-11-22

**Authors:** Elsa Loureiro, Bruna Gomes, João Varajão, Cândida Silva

**Affiliations:** a MIEGSI, University of Minho, Campus de Azurém, Guimarães, Portugal; b Centro ALGORITMI/LASI, University of Minho, Campus de Azurém, Guimarães, Portugal; c Centro ALGORITMI/LASI, CITUR, ESHT, Polytechnic Institute of Porto, Vila do Conde, Portugal

**Keywords:** Information systems, Information technology, Projects, Success, Comparative study, Literature review, Surveys

## Abstract

Information Systems (IS) projects have a long history of negative reputation regarding their success. However, studies that report the success of IS projects are generally focused on software development (which is only one of the many types of IS projects). Furthermore, practically no research compares the success achieved in the different types of IS projects and projects from other areas. Aiming to contribute to filling this gap, this paper presents a critical review of surveys that report the success of IS projects and gives essential recommendations for further studies.

## 1 Introduction

Project success is a core project management concept [1,2]. Due to the importance of this subject, several studies have been carried out over the last years aiming, for example, to identify the factors influencing success or the criteria to evaluate it [3], such as Iriarte and Bayona's [4] or Pereira et al.’s [5] research. Within these studies, it is worth noting the several surveys carried out to report and discuss the success achieved in the projects (e.g., Standish Group [6]; Serrador and Turner [7]; APM [8]).

Regarding Information Systems (IS) projects, a negative outlook has marked their reputation since the first studies by the Standish Group in the 1990s (e.g., Standish Group [6]). In fact, it is commonly thought that these projects fail frequently or have low success levels [9], being remarkable in the path of IS projects the expression “software crisis”. This is penalizing for the area, given that investments in IS are often significant, and they are carried out to cut costs, improve productivity [10,11], increase the quality of services or products [[12], [13], [14]], or improve operational efficiency and long-term performance [15], to mention some of the grounding motivators for conducting IS projects.

However, the questionnaire-based surveys that report on the success of IS projects are generally focused on software development (a type of IS project that typically has the highest failure rates [16]) rather than, for instance, on projects involving organizational interventions (e.g., digital transformation projects). Besides, in several studies, the type of project they are focused on is unclear. Furthermore, practically no research compares the success levels achieved in IS projects and projects from other business areas. That would enable a better understanding of the evolution of success and how IS projects are positioned within the overall project success panorama.

This research aims to help narrow this gap, clarifying the area and type of projects included in research surveys, the success levels obtained, and patterns found. The main research questions are as follows: What success levels have been reported in IS projects? Are these success levels different in IS projects compared to other business areas? How is the success reported in the surveys evaluated?

A literature review was carried out to answer these questions. Ferfolja and Burnett [16] define a literature review as an examination of the research conducted in a specific field of study. Hart [18] adds that it involves the selection of available documents on the topic, which contain information, ideas, data, and evidence. This selection is usually written from a particular standpoint to fulfill specific aims or to express certain views on the nature of the topic and how it is to be researched. In our literature review, we found 28 studies from the last 30 years, which were analyzed in detail.

The contribution to the body of knowledge is twofold. First, the results give a comprehensive perspective of the success achieved in IS projects (taking into account project types, samples, type of participants, geographical focus, success evaluation criteria, and achieved success) and open new avenues for research. Second, analyzing the literature made it possible to identify several weaknesses of the extant research, leading to the proposal of essential guidelines for further research.

This paper is structured as follows. The next section presents the main concepts. The third section describes the research method. The fourth section presents the results and discusses the findings. Finally, the last section presents the main contributions, limitations, and opportunities for further research.

## 2 Background

### 2.1 Information systems projects

Organizations face prominent challenges such as a dynamic business environment [19], stringent competition, continuous technology developments, and a large volume of data [20]. To succeed in tackling these challenges, organizations require efficient IS to remain competitive [21,22]. It is recognized that IS are fundamental for transforming business strategies, business processes, firm capabilities, products, and services, and a key for inter-organizational relationships in extended business networks [23].

As Paul [24] stated, the expression “information systems” is frequently used in discussions and publications, but a common understanding does not even vaguely exist. For some, IS is just Information Technology (IT) or computer applications; for others (e.g., Varajão and Carvalho [25]), it concerns socio-technical systems as an abstraction of the organization itself, focused on the activities and resources that deal with information.

IT handles data and information represented in digital, text, or any other media type and deals with its communication, storage, processing, and printing or exhibition (O'Brien and Marakas [26]). The combination of the use of IT to run the business alongside human decisions that the IT users make leads to the definition of organizational IS.

IT and IS play a central role in contemporary organizations since they are present in almost every aspect of business [27]. In fact, in a rapidly changing technological and business environment, the ability to develop and deploy new systems is an important aspect that can differentiate one organization from another [28]. Moreover, organizations must continuously innovate in terms of product, process, market, and business model to remain sustainable [11], and without IS that is almost impossible.

Overall, an IS is a combination of intelligent agents (human and/or artificial), processes, and IT (hardware, software, and infrastructure) related to the dissemination and use of data, information, and knowledge in an organization [29]. Accordingly, an IS project can be defined as a temporary endeavor undertaken to improve an organizational IS through the use of IT [30,31] and can take on many forms [32], such as digital transformation undertakings [12], IT/IS custom development [33], IS consulting [33], or commercial off-the-shelf application deployment [13].

The management of IS projects is considered a challenging endeavor since they are complex and entail considerable ambiguity and uncertainty [[34], [35], [36]]; as an IS project evolves, goals, priorities, team composition, stakeholder involvement, and contextual factors may change [34,37,38]. Furthermore, IS projects often result in paradigm changes in some sectors, such as financial institutions or banks [39].

Hallows [40] states that a few years ago, IS projects, particularly those that focused on developing new software applications, tended to be monolithic, spanning a year or more before the customer ever saw the results. In recent years, projects have emphasized delivery of interim results, providing value in phases and responding more nimbly to business requirements.

Considering that IS projects vary considerably, the success of every kind of project should be studied in particular.

### 2.2 Dimensions of project success

Project success or failure is difficult to measure and define, as it depends on the stakeholders' perception and perspective [41]. According to Ika [42], there is probably no such thing as “absolute success” in projects. Only “perceived success of a project” exists, and how success is evaluated can change over time. The concept of success is ambiguous, inclusive, and multidimensional, and its definition is related to a specific context. Jugdev and Müller [43] agree that success means different things to different people. The personal objectives of individuals lead them to judge the success of projects differently, and what one person perceives as a successful project can be a failure in another person's eyes [31].

In the Cambridge dictionary [44], success is defined as “the achieving of the results wanted or hoped for” and as “something that achieves positive results”. In the Collins dictionary [45], it is defined as “something that works in a satisfactory way or has the result that is intended” and “the favorable outcome of something attempted”. Lastly, in the Business Dictionary [46], it is defined as “an achievement of an action within a specified period or within a specified parameter”. Success can also mean completing an objective or reaching a goal. Moreover, success can be related to an entire project or be restricted to a single component of a project or task.

It can thus be said that a project is successful when it produces the expected results. Several criteria can be used to evaluate success, but they almost always include meeting requirements on time and within budget and meeting quality requirements [47]. In fact, project success is often measured by the “Iron Triangle” criteria of cost, quality, and time.

Baccarini [1] proposed the Logical Framework Method (LFM) to analyze and understand project success. He distinguished between two concepts: project management success and product success. Project success consists of delivering input and output objectives, and it has three components: meeting time/cost/quality objectives, the quality of the project management process, and satisfying project stakeholders' needs. Product success consists in providing goal and purpose objectives, and it also has three components: meeting the project owner's strategic objectives, satisfying end-users’ needs, and satisfying stakeholders' needs related to the product [48].

Varajão et al. [31] identify several facets of success, including project proposal success, project management success, project success, project outputs success, project outcomes success, project benefits success, project-related operations success, program success, portfolio success, operations success, organizational units success, business success, and society success.

Regarding IS specifically, one of the widely discussed models is DeLone and McLean's IS Success Model [49]. This model consists of six interdependent variables or components for information system success: system quality, information quality, use, user satisfaction, and net impacts. However, it is important to note that this model measures the success of an IS and not the project itself. This is compared to the product success part in Baccarini's [1] model discussed above.

Westhuizen et al. [50] extended the work of Baccarini by identifying the following success dimensions for an information system product: the quality of the project management, whether it is within time and within budget, specified system quality, specified information quality, specified service quality, project stakeholder satisfaction, use of the system, user satisfaction and net benefits (to the organization and others).

Thong et al. [51] have defined project success as the extent to which an IT project actually contributes to achieving business goals. Project managers have a great infiuence on the success of IT projects by performing a multitude of roles according to the project situation. From an IT project manager's perspective, meeting user requirements contributes significantly to IT project success [52,53].

Despite researchers' focus on IT project success, relatively little attention has been given to how individuals attribute project success [54]. How people attribute success individually is likely to have a significant impact on the organizational perception of the final evaluation of a project [55]. Hirschheim and Smithson [56] state that treating IS evaluation, for example, as a technical problem led to meaningless conclusions that overlooked the social activity inherent in the evaluation process. According to Walsham [55], evaluation is a dynamic socio-political process within multi-level social contexts, where personal assessments by stakeholders have a strong infiuence on the evaluation outcomes. It is important, therefore, that those involved in projects can evaluate their contribution in a balanced way.

To sum up, project success is subjective, perceived, and challenging to measure since it is influenced by a variety of factors, including, but not limited to, formal project management practices, skills and competencies of the project manager and other team members, organizational culture, good communication, and support from senior management [41]. Even though the success of project management and the success of the deliverables are not dependent on each other, if project management is not successful, it may jeopardize the success of the deliverables. Thus, the project and its resulting products and services should not be seen in isolation [31,57].

Considering that the surveys focused on studying the success of IS projects have often reported low levels of success, and as evaluating success is unequivocally complex, it is important to look at how the aspects presented in this section have been considered in prior research.

## 3 Method

### 3.1 Literature review

A literature review was carried out to get answers to the research questions. A literature review aims to identify relevant information and outline existing knowledge [17,58]. It is a thorough outline and critical analysis of the relevant available literature on the topic being studied [18,59]. Its goal is to bring up-to-date information about the current literature on a topic and form the basis for another goal, such as the justification for future research in the area. Another purpose is to evaluate and synthesize the information in line with the concepts set for the research [17].

The first step in a literature review is identifying the research question. Ramdhani et al. [58] argue that a sufficiently focused research question is essential before starting a literature review. The next step is identifying and locating appropriate information using well-defined inclusion or exclusion criteria. After that, reading and critically evaluating the information collected is needed. The next step is to file and store the readings and notes. The final step is to plan, organize, and write critically about the collected literature. The literature review should have a logical and coherent structure clearly presented to the reader [17]. Good structuring is essential to enhance the flow and readability of the review [60]. Our study followed these guidelines, and the next sections present the data sources, research strategy, and selection of references.

### 3.2 Data collection

A literature review should contain a clear search and selection strategy [59,61]. Several sources, such as Web of Science (WoS), Scopus, and some ad hoc searching in Google Scholar, were used for this research. To ensure the quality of the information, we focused on papers published in reliable sources, including renowned conferences and journals such as the *Project Management Journal*, the *International Journal of Project Management, the International Journal of Information Systems and Project Management, and the International Conference on Information Systems*.

Some criteria had to be established to select the relevant papers for this study. The inclusion criteria of sources were as follows: to portray a survey reporting project success rates from IS and other areas; published in an academic journal or a conference. In turn, the exclusion criteria were as follows: not written in English or Portuguese (the authors' mother language); published as a preface, editorial, article summary, interview, workshop, panel, or poster. To be considered, a source should comply with all the criteria. There were no temporal restrictions in the search. Search queries and expressions were used on each database. The queries and respective number of results obtained on each database are presented in Appendix A.

The results obtained in the WoS and Scopus search were exported to an Excel document and analyzed individually to avoid excluding relevant papers. After exporting the data of the resulting 24,390 papers, the duplicated ones were removed, leaving 17,802 papers. After reading every paper's title and excluding those clearly unimportant, only 481 were left. Many of the resulting papers focused on success factors and not on reporting the success obtained in projects, so they were excluded (success factors are variables contributing to the likelihood of success; they are not success measures [31]). Next, by reading the paper's abstracts, 68 were selected. From the ad hoc search in *Google Scholar*, only 46 papers were selected (due to the high number of results, only the first five pages of results were considered – even though there were only relevant results until the third page). So, in total, there were 114 references to analyze in full.

Subsequently, all the documents were analyzed, and 62 were selected from all searches as a preliminary paper selection. By analyzing the resulting set in detail, some papers were irrelevant to the study since they did not report success, and some did not present comparable data. So, those had to be discarded, and 28 papers were considered. Table 1 shows the number of papers analyzed by area, organized in the categories “IS”, “other” business area, or, if the project area was not specified in the study, it was considered as projects “unspecified/general”. All the selected papers are presented in Appendix B.

**Table 1:** Projects business areas.

Area	Number of references
IS	23^a^
Other	4^a^
Unspecified/General	4

a three of the references are related to IS and Other business areas at the same time.

Afterward, data was extracted from the final set of papers. As the first step of the data analysis, a codebook was established, including the dimensions for success evaluation, due to the need to synthesize and describe each study. Based on the codebook structure, information was extracted from each paper and summarized and organized in an Excel file. Finally, the data was analyzed in detail, and several graphics and tables were created, which are presented and discussed in the next sections (including data analysis details).

## 4 Findings and discussion

The success reported in the analyzed studies is presented and compared by considering the type of project, type of sample, geographical distribution, and criteria. The next sections also describe the processing that had to be done with the original data to make results comparable. In some studies, there was no information about all the studied aspects, so the number of studies compared in each section varies depending on the aspects reported.

### 4.1 Project success

The studies available in the literature present the success achieved in projects very differently, so it was necessary to define an approach to make the results comparable. The classification used by The Standish Group [6,62] was adopted since it enabled the comparison of the majority of studies’ results, considering three distinct categories. The "successful" category includes projects completed within the initially defined time, cost, and scope. In the "challenged" category, projects have been completed but are over budget, over time, or offer less functionality than initially specified. Categorized as "failure", are the projects canceled during the development cycle or never implemented. Table 2 shows the levels of success reported by each study and the transformations carried out to obtain comparable results.

**Table 2:** Project success rates.

Reference	Project success	Comments
The Standish Group, 1995 [6] (A)	Successful – 16.20 %	Project success (Successful): The project is completed on time and on budget, with all features and functions as initially specified.
	Challenged – 52.70 %	Project challenged (Challenged): The project is completed and operational but over budget, over the time estimate, and offers fewer features and functions than originally specified.
	Failure – 31.10 %	Project impaired (Failure): The project is canceled at some point during the development cycle.
The Standish Group, 2001 [63] (A)	Successful – 28 %	Successful: The project is completed on time and on budget, with all features and functions as initially specified.
	Challenged – 49 %	Challenged: The project is completed and operational but over budget, over the time estimate, and offers fewer features and functions than originally specified.
	Failure – 23 %	Failed (Failure): The project is canceled before completion or never implemented.
Sauer & Cuthbertson, 2003 [64] (A)	Successful – 16 %	The Standish Group categories and definitions were used, and the abandoned projects were considered “failure”.
	Challenged – 75 %	
	Failure – 9 %	
Sonnekus & Labuschagne, 2004 [65] (A)	Successful – 43 %	The Standish Group categories and definitions were used.
	Challenged – 35 %	
	Failure – 22 %	
Sauer et al., 2007 [66] (B)	Successful – 67.70 %	The projects were categorized into five performance types: abandoned projects, budget-challenged, schedule-challenged, good performers, and star performers. Abandoned projects were considered “Failure” (38); Projects with budget (21) or schedule (74) challenged were considered “Challenged”; Good performers (249) and Star performers (30) were considered “Successful” projects.
	Challenged – 23.10 %	
	Failure – 9.20 %	
The Standish Group, 2007 [67] (A)	Successful – 35 %	Successful: The project is completed on time, on budget, and met user requirements. Challenged: The project has cost or time overruns or did not fully meet the user's needs.
	Challenged – 46 %	
	Failure – 19 %	
Emam & Koru, 2008 [68] (B)	Successful – 51.50 %	Project success is measured based on five criteria (user satisfaction, meeting schedule and budget targets, product quality, and team productivity) and a four-point Likert scale (excellent, good, fair, and poor). The authors present the values for “Successful” or “Failure” projects as an interval, so it was considered the average of those intervals for comparison purposes: Successful = (48 + 55)/2; Failure = (17 + 22)/2
	Challenged – x	
	Failure – 19.50 %	
Varajão et al., 2008 [69] (C)	Budget – 85.53 %	Only the percentage of success achieved in each criterion is shown.
	Scope – 72.37 %	
	Time – 63.82 %	
Maglyas et al., 2010 [70] (B)	Successful – 29 %	The authors considered a project successful if it had finished without budget and time overhead (totally successful) or finished with time overrun only. For comparison purposes, we considered “Successful” only the projects without overruns.
	Challenged – 71 %	
	Failure – x	
Alfaadel et al., 2012 [48] (A)	Successful – 47.57 %	According to The Standish Group definitions, respondents decided how many projects were successful, challenged, or impaired. The total number of successful (2613), challenged (2017), and impaired (863) projects were presented. Then, the percentage of each category was calculated by dividing these values by the total number of projects (5,493). Impaired projects were considered “Failure”.
	Challenged – 36.72 %	
	Failure – 15.71 %	
Marnewick, 2012 [57] (B)	Successful – 59 %	Respondents were asked to indicate how many projects failed, were challenged, and were successful in the last two years, with the definitions of failed, challenged, and successful being left open to interpretation.
	Challenged – 29 %	
	Failure – 12 %	
Pretorius et al., 2012 [41] (A)	Successful – 46 %	Successful: A project that is delivered on time, within budget, within scope, and complies with the quality requirements.
	Challenged – 36 %	Challenged: A project that is completed but is late, over budget, or does not meet all the requirements.
	Failure – 18 %	Failed (Failure): A project that is never completed or does not meet customer requirements.
The Standish Group, 2013 [71] (A)	Successful – 39 %	Successful: Delivered on time, on budget, with required features and functions.
	Challenged – 43 %	Challenged: Late, over budget, and/or with less than the required features and functions.
	Failure – 18 %	Failure: Canceled prior to completion or delivered, and never used.
Varajão et al., 2014 [72] (C)	Software projects	This article only reports the percentage of how much each criterion was met in each industry.
	Cost – 72 %	
	Time – 59 %	
	Scope – 70 %	
	Construction projects	
	Cost – 70 %	
	Time – 72 %	
	Scope – 88 %	
Association of Project Management, 2015 [8] (B)	Successful – 72 %	The authors used four categories (wholly successful, very successful, moderately successful, and unsuccessful) to describe the projects' success levels. The categories of wholly successful and very successful were merged and were considered “Successful” projects (22 + 50 = 72). Moderately successful was considered the “Challenged” category (22 %), and unsuccessful was the “Failure” category (6 %).
	Challenged – 22 %	
	Failure – 6 %	
Serrador & Turner, 2015 [7] (B)	Successful – 49.35 %	The total number of very successful (233), successful (451), mixed (345), not fully successful (259), and failure projects (98) were presented. Then, the percentage of each category was calculated by dividing these values by the total number of projects (1,386). The categories that were not fully successful and mixed were merged and considered as the “Challenged” category. The categories of successful and very successful were considered as the “Successful” category.
	Challenged – 43.58 %	
	Failure – 7.07 %	
The Standish Group, 2015 [62] (A)	Successful – 36 %	The study presents a traditional and a modern approach. The traditional definition of success is on time, on budget, and on target, which means the project was resolved within a reasonable estimated time, stayed within budget, and contained a good number of the estimated features and functions. The modern definition of success is on time, on budget, and with a satisfactory result, which means the project was resolved within a reasonable estimated time, stayed within budget, and delivered customer and user satisfaction regardless of the original scope. In order to compare the success of the projects, the traditional approach was used since this is the approach used in almost all the remaining studies.
	Challenged – 45 %	
	Failure – 19 %	
Donkersloot & Gaikema, 2018 [73] (B)	Successful – 63.18 %	Successful projects include projects that were completed on time, on budget, and on target. Only information about successful projects was provided. The success of small and large projects is presented, and the average weight of the two types of projects is considered for global success: (75 % ∗ 90 + 10 % ∗ 20)/110
	Challenged – x	
	Failure – x	
Frączkowski et al., 2018 [74] (C)	Project budget – 38.80 %	This article has different categories for each success criterion; therefore, only the rates related to the success of the projects were considered due to the non-compatibility of the results. In the project scope criteria, the “almost 100 %” category was considered “Successful”, and in the case of the customer satisfaction criteria, the high category was considered “Successful”.
	Project time – 51.20 %	
	Project scope – 56.40 %	
	Project manager satisfaction – 49.40 %	
	Customer satisfaction – 17.50 %	
The Standish Group, 2018 [75] (A)	Successful – 36 %	The study provided a "traditional", a "modern" and a “pure success” approach. The modern definition of success is on time, on budget, and with a satisfactory result. The traditional assessment is on time, on budget, and on target. The pure success approach is the combination of customer satisfaction with return on value to the organization. To compare the success of the projects, the traditional approach was used since this is used in almost all the remaining studies.
	Challenged – 45 %	
	Failure – 19 %	
Hoxha & McMahan, 2019 [76] (B)	Successful – 44.40 %	To measure project success, it was used the project implementation profile (PIP) proposed by Slevin and Pinto (PIP uses 12 specific questions to evaluate success). Success was presented on a scale from 0 to 100, and in the article, it was considered a “Successful” project when the success level was more than 69.
	Challenged – x	
	Failure – x	
Laranjeira et al., 2019 [77] (B)	Successful – 61.70 %	Project success is presented on a scale from 0 to 7, where 0 means "does not know", 1 means "total failure", and 7 means "total success". Levels 6 and 7 were considered as “Successful”. When presenting the results, level 1 was merged with other levels, so it was not possible to identify the percentage of challenged and failed projects.
	Challenged – x	
	Failure – x	
Khoza & Marnewick, 2020 [78] (B)	Successful – 64 %	Only information about the average success rate of the projects is shown, taking into account five groups of success evaluation criteria: process success, project management success, deliverable success, business success, and strategic success.
	Challenged – x	
	Failure – x	
Varajão et al., 2020 [79] (B)	Successful – 6.90 %	The authors used four categories (total success, successful, unsuccessful, and failure) to describe the projects' success levels. The categories successful (4.60 %) and total success (2.30 %) were merged and considered “Successful”. The categories of unsuccessful (28.74 %) and failure (4.60 %) projects were considered “Failure”. 59.77 % of participants did not answer.
	Challenged – x	
	Failure – 33.34 %	
Bilir & Yafez, 2021 [80] (A)	Successful – 48.13 %	Successful: The project is completed on time, on budget, and with the predefined scope; Challenged: The project is completed but over budget, over the estimate, or without some initially defined functionalities; Failed (Failure): The project is canceled before completion, or the customer has never used its output.
	Challenged – 45 %	
	Failure – 6.87 %	
Varajão et al., 2021 [29] (B)	Successful – 52.10 %	Success is presented on a scale from 0 to 10, where 0 means a project abandoned, and 10 menas a complete success. When presenting the results, level 0 was merged with other levels, so it was impossible to identify the percentage of challenged and failed projects. Levels 9 and 10 were considered “Successful”.
	Challenged – x	
	Failure – x	
Pereira et al., 2022 [5] (B)	Successful – 27.85 %	Project success is presented on a scale from 0 to 10, where 0 means the project's abandonment, and 10 means a total success. Levels 9 and 10 were considered “Successful”.
	Challenged – x	
	Failure – x	
Pretorius et al., 2022 [81] (B)	Successful – 70 %	–
	Challenged – 22 %	
	Failure – 8 %	

In order to make a consistent and rigorous comparison of results, the articles were divided into three groups. Group A includes articles that present the success of projects according to The Standish Group's approach [6,62], taking into account only the criteria of time, cost, and scope. Group B includes articles that do not limit the evaluation criteria to the Iron Triangle criteria, do not specify the criteria to evaluate project success, or only present the success achieved in the projects without detailing the challenged or failed projects. Group C contains articles that do not present the overall success achieved by the projects but only identify the success achieved in certain criteria, so they will only be analyzed in detail in the section that focuses on the success achieved in each criterion. Table 3 shows the articles in the groups mentioned to make it easier to understand how they are analyzed in the following sections.

**Table 3:** Segmentation of articles for analysis.

Group	Article
**Group A** Studies that present the success of projects according to the categories and criteria of The Standish Group	[6] The Standish Group. (1995). The CHAOS Report. *The Standish Group*.
	[63] The Standish Group. (2001). Extreme Chaos 2001. *The Standish Group*.
	[64] Sauer, C., & Cuthbertson, C. (2003). The State of IT Project Management in the UK 2002–2003. *Computer Weekly Project/Programme Management Survey*, 1–82.
	[65] Sonnekus, R., & Labuschagne, L. (2004). Establishing the relationship between IT Project Management Maturity and IT Project Success in a South African context. *Proceedings of the 2004 PMSA International Conference ‘Global Knowledge for Project Management Professionals’*, 183–192.
	[67] The Standish Group. (2007). Standish Group Report: There's Less Development Chaos Today. *The Standish Group*.
	[48] Alfaadel, F., Alawairdhi, M., & Al-Zyoud, M. (2012). Success and failure of IT projects: a study in Saudi Arabia. 11th *WSEAS International Conference on Applied Computer and Applied Computational Science*, 77–82.
	[41] Pretorius, S., Steyn, H., & Jordaan, J. C. (2012). Project Management Maturity and Project Management Success in the Engineering and Construction Industries in Southern Africa. *South African Journal of Industrial Engineering*, *23*(3), 1–12.
	[71] The Standish Group. (2013). CHAOS Manifesto 2013. *The Standish Group*.
	[62] The Standish Group. (2015). Chaos Report 2015. *The Standish Group*.
	[75] The Standish Group. (2018). Decision latency theory: it is all about the interval. *The Standish Group*.
	[80] Bilir, C., & Yafez, E. (2021). Project success/failure rates in Turkey. *International Journal of Information Systems and Project Management*, *9*(4), 24–40.
**Group B** Studies that only present the overall success of the projects or use criteria for evaluation different from The Standish Group's	[66] Sauer, C., Gemino, A., & Reich, B. H. (2007). The impact of size and volatility on IT project performance. *Communications of the ACM*, *50*(11), 79–84.
	[68] Emam, K. el, & Koru, G. A. (2008). A replicated survey of IT software project failures. *IEEE Software*, *25*(5), 84–90.
	[70] Maglyas, A., Nikula, U., & Smolander, K. (2010). Comparison of two models of success prediction in software development projects. 6th *Central and Eastern European Software Engineering Conference*, 43–49.
	[57] Marnewick, C. (2012). A longitudinal analysis of ICT project success. *ACM International Conference Proceeding Series*, 326–334.
	[8] Association of Project Management. (2015). Conditions for Project Success: APM research report. *APM Conference*.
	[7] Serrador, P., & Turner, R. (2015). The Relationship Between Project Success and Project Efficiency. *Project Management Journal*, *46*(1), 30–39.
	[73] Donkersloot, M., & Gaikema, M. (2018). An international research on IT-projects, to leverage success of Dutch IT-projects. *Informagency*, 1–4.
	[76] Hoxha, L., & Mcmahan, C. (2019). The Influence of Project Manager's Age on Project Success. *Journal of Engineering, Project, and Production Management*, *9*(1), 12–19.
	[77] Laranjeira, M., Trigo, A., & Varajão, J. (2019). Success of Software Development Projects in Portugal - preliminary results. *19.*^*a*^*Conferência Da Associação Portuguesa de Sistemas de Informação (CAPSI’2019)*.
	[78] Khoza, L., & Marnewick, C. (2020). Waterfall and Agile information system project success rates - A South African perspective. *South African Computer Journal*, *32*(1).
	[79] Varajão, J., Trigo, A., Moura, I., & Pereira, J. L. (2020). IS Project Management Success in Developing Countries. *European, Mediterranean, and Middle Eastern Conference on Information Systems*, *402*, 769–780.
	[29] Varajão, J., Pereira, J. L., Trigo, A., & Moura, I. (2021). Information systems project management success. *International Journal of Information Systems and Project Management*, *9*(4), 62–74.
	[5] Pereira, J., Varajão, J., & Takagi, N. (2022). Evaluation of Information Systems Project Success – Insights from Practitioners. *Information Systems Management*, *39*(2), 138–155.
	[81] Pretorius, S., Bond-Barnard, T. J., Steyn, H., & Jordaan, J. (2022). Project Success and Project Manager Competency. *Portland International Conference on Management of Engineering and Technology (PICMET)*.
**Group C** Studies that only present the success achieved in each evaluation criterion	[69] Varajão, J., Cardoso, J., Gonçalves, D., & Cruz, J. B. (2008). Análise à gestão de projetos de desenvolviemnto de software em grandes empresas portuguesas. *Semana Informática*, *904*, 10–12.
	[72] Varajão, J., Dominguez, C., Ribeiro, P. M. G. de A., & Paiva, A. (2014). Failures in software project management – are we alone? A comparison with construction industry. *The Journal of Modern Project Management*, *2*(1), 23–25.
	[74] Frączkowski, K., Gładysz, B., Kuchta, D., & Stanek, S. (2018). Selection of IT projects to be implemented in an organisation to maximise their success probability. *Journal of Decision Systems*, *27*(1), 111–122.

**Fig. 1:**
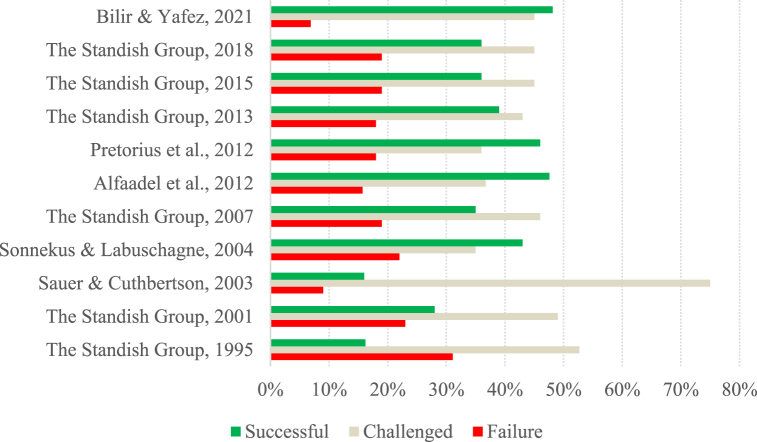
Success rate per study (Group A).

**Fig. 2:**
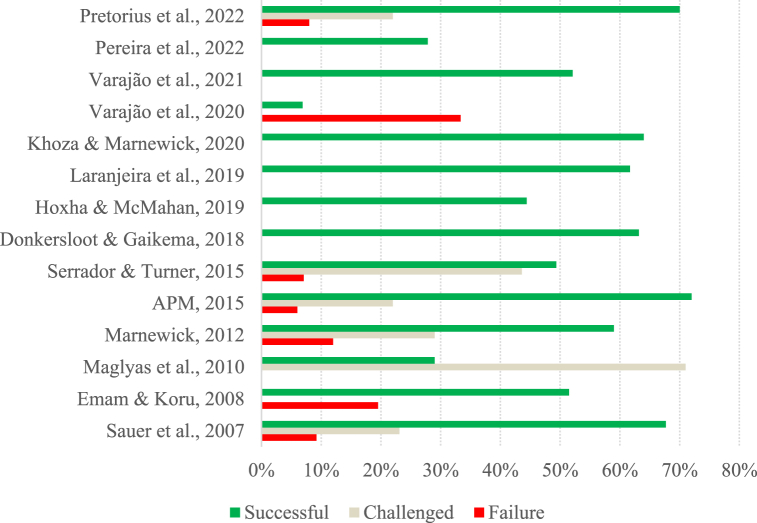
Success rate per study (Group B).

To allow a more detailed analysis of the data presented in Table 2, two charts (Fig. 1, Fig. 2) were created with the success rates reported in the studies. Considering only the articles belonging to group A, the low success rates achieved by the projects over the years are quite notorious. Although Group A comprises studies carried out over more than two decades, between 1995 and 2021, they all show success rates lower than 50 %.

When we look at The Standish Group's studies in isolation, we can see that they have lower success rates than the other studies. While The Standish Group's studies have success rates of 39 % or less, the other studies have success rates of over 40 %, except for the study by Sauer and Cuthbertson [64], which has a success rate of 16 %. Note that the high occurrence of The Standish Group's studies in Group A can bring some bias to the results.

Concerning the evolution of project success over the years, in The Standish Group's studies, there is a positive evolution in project success, from lower success rates in the early studies, followed by stabilization, with around a third of projects being presented as successful since 2007. In the remaining studies, the success rate of projects has been stable over the years, with figures of around 46 %. Again, the exception is the study by Sauer and Cuthbertson [64], which shows a success rate of 16 %, a figure similar to that presented by The Standish Group [6].

The rate of challenged projects is between 35 % and 52.70 %, with the Sauer and Cuthbertson [64] study showing a very high rate of challenged projects compared to the others (75 %). Thus, the average rate of challenged projects is 46 %, which is higher than that of successful projects. This can be explained by the fact that the success of projects is measured in these studies by considering the project's initial planning. Project failure rates were between 6.87 % and 23 %, except for the study by The Standish Group [6], which showed a failure rate of 31.10 %.

The studies in group B have a more optimistic perspective regarding project success, with 64 % reporting project success rates of over 50 %. Despite this, the success rate of the projects varies greatly from study to study, ranging from 6.90 % to 72 %. The study by Varajão et al. [79] shows the lowest success rate (6.90 %). Still, it can be explained by the fact that it was a study carried out in developing countries in which 59.77 % of respondents could not indicate the success achieved in the project they were reporting [79].

In this sense, we can see that, in general, success rates may vary considerably from study to study, and in Group A, there has been a noticeable stabilization over the last few years. The results of the studies belonging to Group A reinforce the idea of Pretorius et al. [81] and Varajão et al. [29], who describe IS projects as problematic and with lower-than-expected success rates. Furthermore, although the studies belonging to Group B have more satisfactory success rates than those in Group A, they still have much room for improvement.

### 4.2 Project types

Upon analyzing all the studies, four categories of projects were identified: Software development, IS in general, projects in general, and others.

Most software project category studies used the terminology "software projects" and "software development projects", thus being included in the category “software projects”. Regarding IS projects, studies that mentioned "IT projects" and/or "IS projects" fell into this category since the terms IT and IS are often used indistinctly. The “projects in general” category includes the studies that did not specify the types of projects reported, along with the studies that mentioned "projects in general". Finally, the “other projects” category refers to projects in specific areas that do not fall into the software development/IS categories, and although some of these studies do not explicitly mention the project area, it is worth highlighting the prominence of construction projects [41,72] in this category.

Finally, it is also worth mentioning that, of the selected studies, some report the success of projects belonging to two different categories, two of which refer to IS projects and others (see Bilir and Yafez [80] and Varajão et al. [79]), and the other one to software projects and others (see Varajão et al. [72]).

**Fig. 3:**
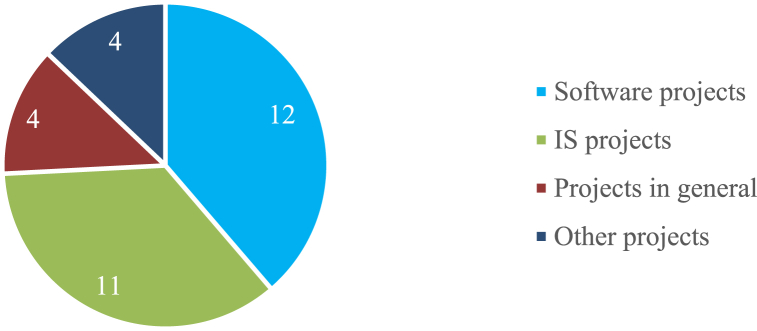
Project type.

The chart in Fig. 3 presents the number of studies classified in each category.

**Fig. 4:**
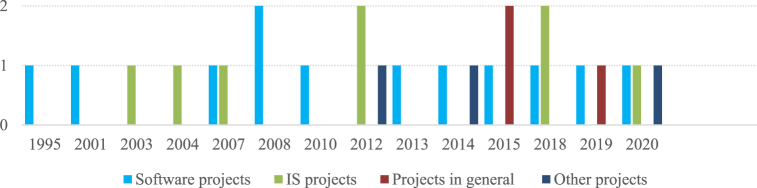
Number of papers by year.

By observing Fig. 3, it is possible to conclude that software development projects and IS in general are the most reported regarding project success. As software development projects are a specific type of IS project, they are by far the most studied area for project success. In fact, other business areas rarely report on the success of their projects. Fig. 4 shows the number of project types by year.

Three studies report the success of more than one type of project, but only one has been included in this analysis. In the study by Varajão et al. [79], only the joint success of the various types of projects is reported, while in the case of Varajão et al. [72], although success is distinguished according to the type of project, it only presents the success achieved in specific criteria, so they cannot be considered in this section. The study by Bilir and Yafez [80] includes both IS projects and other projects. However, it was included in the analysis because it presents success rates separately according to the project type, allowing the results to be compared.

**Fig. 5:**
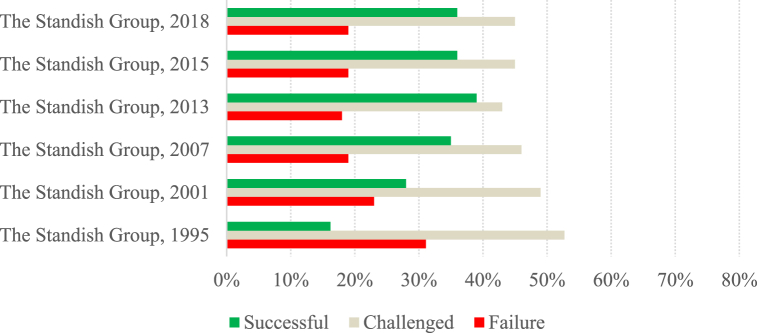
Success of software projects (Group A).

**Fig. 6:**
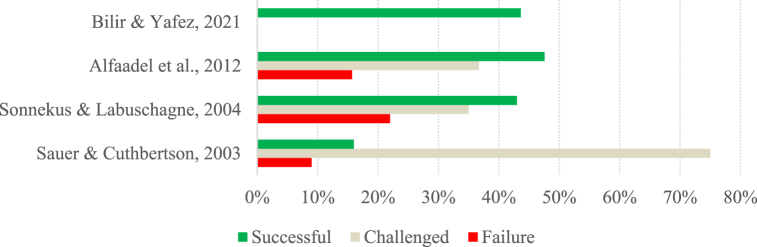
Success of IS projects (Group A).

**Fig. 7:**
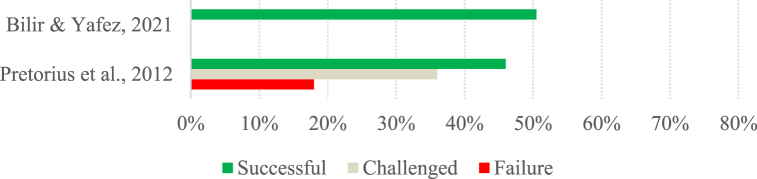
Success of other projects (Group A).

**Fig. 8:**
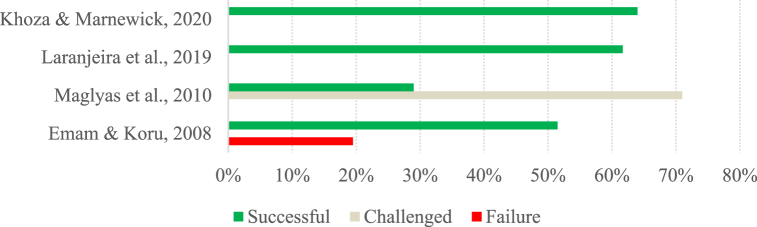
Success of software projects (Group B).

**Fig. 9:**
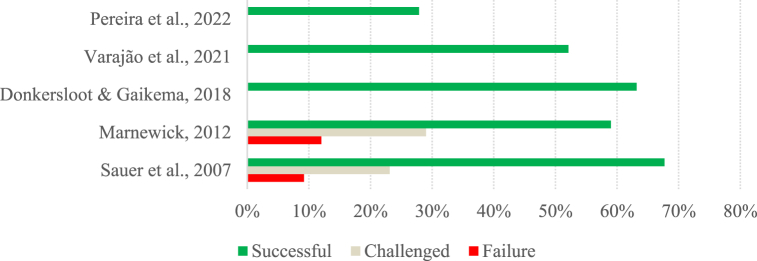
Success of IS projects (Group B).

**Fig. 10:**
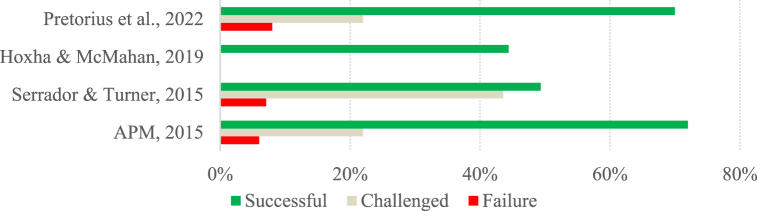
Success of projects in general (Group B).

Fig. 5, Fig. 6, Fig. 7, Fig. 8, Fig. 9, Fig. 10 show the project success rates by project type.

When comparing the success rates of group A projects considering the project type, we notice that software projects have relatively similar success rates to IS projects (see Fig. 5, Fig. 6). Since software projects are a specific type of IS project, if we consider the category of IS projects as a whole, we see that, on average, it has similar success rates to other projects (Fig. 7). However, only two studies were analyzed in the latter category. Thus, the studies of IS projects, in general, provide an overview of project success over the years, while the studies of other projects only focus on the years 2012 and 2021. Comparing the studies of other projects and IS projects for the same year, it can be seen that the 2012 studies have similar success, with a slight difference being found in the success reported by the 2021 studies, where projects in other areas stand out positively.

Focusing on the studies in group B, we see that the success rates for the different types of projects are relatively similar and, on average, close to 54 %. Interestingly, in this group, the lowest success rates (around 28 %) are found in software and IS projects, and the highest success rates (around 70 %) concern projects in general.

Overall, we cannot say that projects in other areas are more or less successful than IS projects since the number of studies reporting on the success of IS projects is quite different from the number of studies reporting on projects in other areas, as only two of the studies dealing with other projects meet the requirements to be analyzed in this section. In the studies belonging to group A, there is a noticeable initial positive trend followed by a stabilization of the success of both software and IS projects, although they still have relatively low success rates. In the case of the studies in group B, there is no discernible pattern in the evolution of success rates over time in any project type.

### 4.3 Samples

A significant discrepancy is noticeable when comparing the studies’ data regarding the number of participants and projects, with numbers varying from the dozens to the thousands. Most of the studies reported the number of participants and projects, nine studies presented only the participants, three studies reported the number of projects, and five studies reported neither. Table 4 shows the number of participants and the number of projects per study.

**Table 4:** Sample sizes.

Reference	No. of participants	No. of projects
Sauer & Cuthbertson, 2003 [64]	1456	421
Marnewick, 2012 [57]	1067	–
APM, 2015 [8]	862	–
Serrador & Turner, 2015 [7]	859	1386
Sonnekus & Labuschagne, 2004 [65]	819	1633
Khoza & Marnewick, 2020 [78]	617	617
Pretorius et al., 2022 [81]	600	9389
Sauer et al., 2007 [66]	412	–
Emam & Koru, 2008 [68]	388	–
The Standish Group, 1995 [6]	365	8380
Alfaadel et al., 2012 [48]	308	5493
Pretorius et al., 2012 [41]	255	273
Laranjeira et al., 2019 [77]	193	–
Hoxha & McMahan, 2019 [76]	108	–
Varajão et al., 2021 [29]	107	472
Varajão et al., 2020 [79]	87	–
Frączkowski et al., 2018 [74]	80	80
Varajão et al., 2014 [72]	80	–
Pereira et al., 2022 [5]	64	158
Varajão et al., 2008 [69]	40	–
Bilir & Yafez, 2021 [80]	–	320
Donkersloot & Gaikema, 2018 [73]	–	110
Maglyas et al., 2010 [70]	–	48

To ease the analysis of the collected data, the studies were divided into values over 500 and under 500. When observing the table, it is possible to conclude that studies mostly include fewer than 500 participants or projects. In addition, they more often report the number of participants rather than the number of projects, which is curious given that the study's object of interest is the project's success.

Focusing on the studies belonging to group A, a similar average success rate is seen when the number of participants or projects is less than or greater than 500. In the case of studies belonging to group B, there is a higher average success rate in studies that include more than 500 participants or projects compared to studies that include smaller samples.

Regarding the type of sample, no relevant pattern was noticeable in any of the groups, with the average success rates being relatively similar in cases where only the number of participants, the number of projects, or both are presented.

### 4.4 Type of participants

The study sample is a crucial aspect to consider since it may bias results. However, several studies do not report participants with the required details. Table 5 shows the type of participants for each study. When no detail is given about the percentage of participant types, this is marked with an “X” (if there is only a type of participant, the “X” represents 100 %). Those who had no information regarding the participants are in the “no information” category, and the types of participants who were only mentioned in one study are in the “other” category.

**Table 5:**
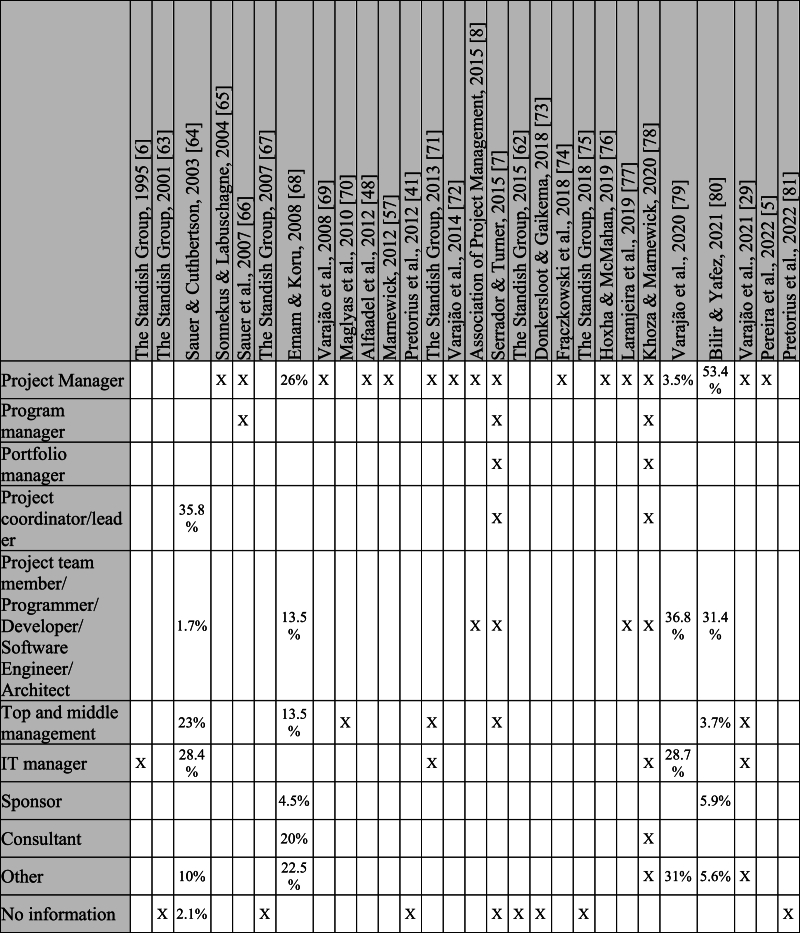
Type of participants.

It is also important to note that the studies use different and very diverse terms to identify the participants, generally not describing them, which makes it difficult to group and compare.

It should be noted that the study by Emam and Koru [68] stated that there were 388 responses, but presented only information regarding the type of participants for 200, so the percentages on the table were calculated based on those 200. Incomplete data on participant types can bias results and potentially misrepresent the true characteristics of the study population.

Table 5 shows that project managers are the main focus of studies reporting on project success, followed by the project team, middle and top managers, and IT managers. It is also possible to observe that some studies focus on various types of participants, which makes it possible to understand the different perspectives of the people involved in projects and make results more trustable.

### 4.5 Geographical focus

**Fig. 11:**
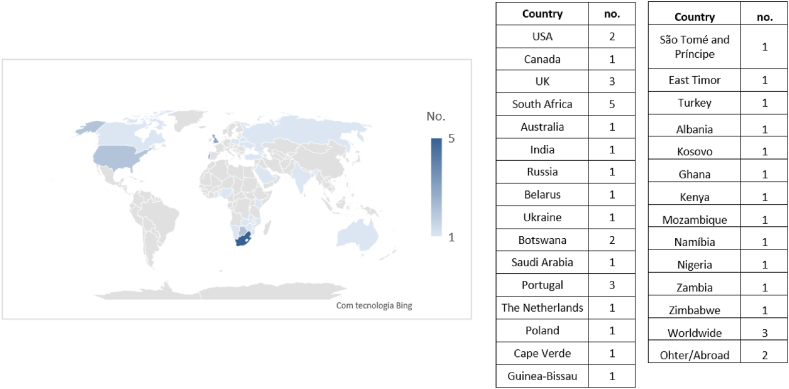
Geographical distribution of the samples.

The map in Fig. 11 shows the origin of the data reported in the studies, with some studies addressing more than one country. By analyzing the map, it is possible to see the number of studies that focused on each country, and it is notorious for a wide geographical spread of studies, although they focus particularly on countries such as South Africa, the United Kingdom, and Portugal.

When trying to understand the success reported in studies based on their geographical distribution, it is important to take into account that some studies report projects from different countries, so the success presented may not represent the reality of some of the countries mentioned and thus cause a certain bias in the results. With this in mind, this section only considered ten articles that reflected the success rates of projects in just one country.

The studies with the lowest success rates fall into group A and are based on projects from the United Kingdom and the United States, with a success rate of 16 % [64] and 28 % [63] (both studies with more than 20 years), respectively. The remaining studies in group A concern countries such as South Africa, Saudi Arabia, and Turkey, which have between 40 % and 50 % success rates. In group B, two studies reflect the success achieved in projects in South Africa in 2012 and 2020, with similar success rates of around 60 %. The remaining studies in group B have success rates of between 60 % and 70 % and concern countries such as the Netherlands, Portugal, and the United Kingdom.

Considering the geographical distribution, there are no observable relevant patterns on the success achieved in the projects since the studies are scattered worldwide and most countries are addressed only once (in one study).

### 4.6 Success evaluation criteria

The criteria used to evaluate success in each study are presented in Table 6. Some studies do not contain this information and were grouped in the "not specified" category. The criteria that were mentioned only once were included in the "other" category.

**Table 6:**
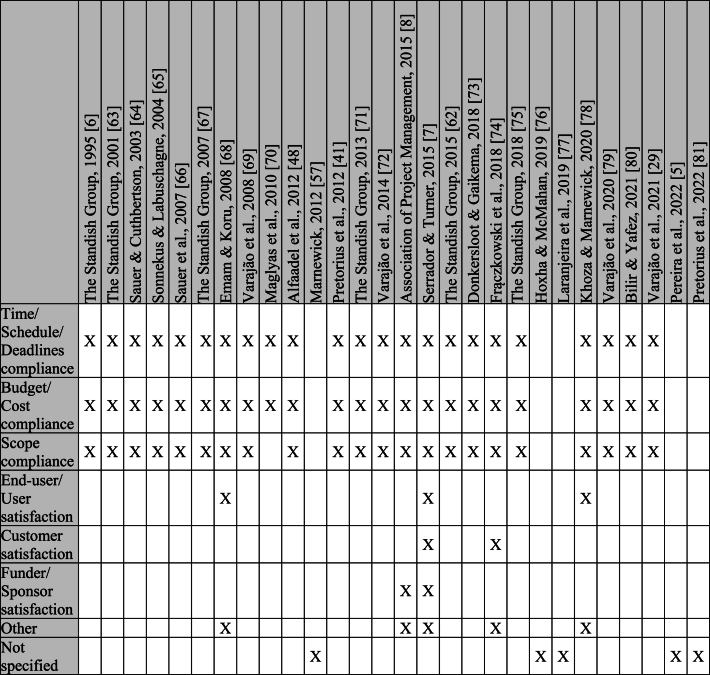
Success evaluation criteria.

Table 6 shows the high importance of the Iron Triangle criteria (compliance with time, cost, and scope). These criteria were used in practically all the studies (that specified the criteria used). Furthermore, of these studies, only five considered other criteria when evaluating success (e.g., user satisfaction, client satisfaction, and sponsor satisfaction).

Overall, the success rates for the Iron Triangle criteria are relatively satisfactory, with average success rates around 60 % for each criterion. In addition, it is notable that at least half of the studies that show success in meeting the cost and scope criteria have success rates of over 70 %, which is a relatively high value.

The studies of Varajão et al. [79] and Varajão et al. [29] present the success rates of compliance with time, cost, and scope criteria, considering the “compliance” with the criteria “without changes” and “with changes compared to the initial planning”. In this sense, it is possible to observe that the compliance rate of each criterion taking into account changes is higher than the compliance rate of each criterion when the initial planning is taken into account for evaluating success (except for compliance with the budget in the study by Varajão et al. [29]). With the ever-changing environment and the uncertainty associated with IS projects, requirements are expected to change throughout the project cycle. In this sense, it becomes relevant that the evaluation of the project considers meeting the requirements considering the evolution of customer needs (and not only the initial requirements).

Although the success rates achieved in each criterion are generally positive, note that approximately 70 % of studies reporting overall success rates of projects below 50 % do not specify the success rates achieved in each of the criteria or do not specify the criteria used in the evaluation. The information presented in this section does not reflect those cases, which may influence the results.

Overall, the average success in studies that evaluate success with more criteria than the Iron Triangle is higher. In addition, we can observe that all studies that have success rates below 30 % only use the criteria of the Iron Triangle in assessing the success of projects or do not specify the criteria used, which may lead to the conclusion that strictly considering the original criteria and definitions used by The Standish Group [6] is likely to result in a lower rate of successful projects.

## 5 Conclusion

### 5.1 Main insights

Some important considerations can be made upon analyzing the data collected from all the studies. Over time, the evaluation of the success of projects has been presented in a very diverse way in the various studies, making it difficult to compare results. However, some patterns could be established after adjusting the data and segmenting it into two groups (A and B).

Group A projects, considering the Iron Triangle as the sole evaluation criteria, reveal low rates of successful projects and significant rates of challenged projects. The projects belonging to group B present a more optimistic perspective toward success, although they still have a good improvement margin. Thus, following only the criteria and the classical definitions by The Standish Group will most likely result in a lower percentage of successful projects, eventually not reflecting reality correctly.

The studies represent mostly software and IS projects in general; despite the discrepancy in the number of articles reporting these projects compared to those reporting projects from other areas, no significant differences were found.

Regarding the sample size, the studies are based on samples of very diverse dimensions, although they have mostly on less than 500 participants and projects. In group A, there is no noticeable variation in success when considering the sample size, while in group B, there are notoriously higher success rates in studies with larger samples. Since sample size may impact the results' reliability, researchers should struggle for larger samples whenever possible.

Regarding the sample participants, some studies focus only on project managers, which can lead to biased results. A diverse sample of project stakeholders would allow for the covering of various perspectives and interests. Moreover, some studies analyze the success of projects based on perceptions and estimates. This means the data can be ambiguous and not factual, compromising the results. Future studies should address this issue by including diverse participants to ensure a comprehensive analysis.

Regarding the criteria for assessing success, the criteria of the Iron Triangle (fulfillment of time, cost, and scope) stand out. Although only a few studies are based on criteria other than the Iron Triangle criteria, success tends to be higher when more criteria are considered. The Iron Triangle provides a comparable picture of success, but the credibility of success reporting is improved when success is evaluated with a richer set of criteria [82].

It is also noticeable that most studies do not have enough details about some analysis dimensions. In future studies related to the evaluation of success, all dimensions analyzed should be described in detail, from the demographics of respondents to each criterion meaning.

### 5.2 Implications for research and practice

This study makes a significant contribution to the body of knowledge in three ways. Firstly, it provides a comprehensive perspective of the success achieved in IS projects based on the results of survey studies from the last 30 years, taking into account project types, samples, participants, geographical focus, evaluation criteria, and achieved success. This raises awareness of IS project success and paves the way for future research. Secondly, the literature analysis revealed several weaknesses in existing research, which this study addresses by proposing crucial guidelines for future studies.

The findings led to important recommendations to improve questionnaire-based future surveys focused on project success. Thus, new studies should.

- Describe in detail the methodology for collecting data and analyzing results;
- Present detailed information about the sample (e.g., participants' demographics) – it would be commendable to have participants other than the project managers so the results can be richer and unbiased;
- Identify and describe the type of projects that are being studied since most available studies only present vague information;
- Clearly define the criteria used to evaluate success – use a rich set of criteria, but always include the common basic criteria (e.g., the Iron Triangle criteria) so that results can be compared;
- Whenever possible, focus not only on the project management but also on the post-project results;
- Each respondent should report success by project individually, and the contextual information should be presented in full (for instance, how the success of projects was evaluated and by whom).

By raising awareness of the common features and missing details (e.g., sample participants, type of project, evaluation criteria, etc.) of studies, we not only provide new fundamental theoretical insights but also expect to contribute to more and improved research in this crucial area.

Thirdly, for practitioners, the cumulative knowledge from the surveyed studies offers a broad picture of IS success, including a better understanding of project success dimensions, criteria, and guidelines for evaluation (e.g., use of a rich set of criteria for evaluating success). Furthermore, the presented findings can be used as a benchmark for project success.

### 5.3 Limitations and future research directions

Some challenges and limitations were encountered when developing this study.

There is a clear lack of uniformity between the studies, making the analysis and comparison difficult. The shortage of detail in several studies is also a significant issue, including the absence of information regarding the data collection, the sample, and how the reported findings were achieved, among other related issues encountered in many studies. Due to the lack of uniformity of the data collected and reported results, some processing had to be made to the data of several studies. Further studies should include detailed descriptions of these aspects to make possible analysis with minimum processing of secondary data.

Considering that there were only 28 studies suitable for comparison, grouped into generic categories (software projects, IS projects, projects in general, and other projects), this limits the generalization of findings across all types of IS projects or other business areas and demands new focused research. In other words, new studies are needed to focus on the success of different types of projects and business areas.

Notably, we could only include in our research studies from a limited set of countries, most of them with only one study. This also calls for more contributions, not only on still uncovered geographies but also replication studies that should be carried out periodically to identify the evolution of the success of IS projects over time.

Finally, the success of projects can be influenced by several factors [31], including changes in technology, market conditions, project management methodologies, or organizational culture, to name a few. Further studies should explore such factors and their direct and indirect relation with success since they could provide important insights for improving the results of projects.

## CRediT authorship contribution statement

**Elsa Loureiro:** Writing – original draft, Visualization, Validation, Investigation, Formal analysis, Data curation. **Bruna Gomes:** Writing – original draft, Visualization, Validation, Investigation, Formal analysis, Data curation. **João Varajão:** Writing – review & editing, Visualization, Validation, Supervision, Project administration, Methodology, Investigation, Funding acquisition, Data curation, Conceptualization. **Cândida Silva:** Writing – review & editing, Validation, Supervision, Methodology.

## Ethics declaration

This study did not need a review and/or approval by an ethics committee because it did not involve human participants.

## Data availability statement

No primary empirical data was used for the research described in the paper.

## Declaration of competing interest

The authors declare that they have no known competing financial interests or personal relationships that could have appeared to influence the work reported in this paper.

## Figures and Tables

**Table:** 

Database	Query	Results
WoS	(project or program or programme or portfolio or pmo) AND (success) AND (survey or questionnaire)	9477
Scopus	(project or program or programme or portfolio or pmo) AND (success) AND (survey or questionnaire)	14,913
Ad hoc	(“project success”) AND (survey AND questionnaire)	1,482,000
	comparative AND study AND “project success”	1,516,700
	“project success rate”	469,400

**Table:** 

Business Area	Reference
Information Systems	[6] The Standish Group. (1995). The CHAOS Report. *The Standish Group*.
	[63] The Standish Group. (2001). Extreme Chaos 2001. *The Standish Group*.
	[64] Sauer, C., & Cuthbertson, C. (2003). The State of IT Project Management in the UK 2002–2003. *Computer Weekly Project/Programme Management Survey*, 1–82.
	[65] Sonnekus, R., & Labuschagne, L. (2004). Establishing the relationship between IT Project Management Maturity and IT Project Success in a South African context. *Proceedings of the 2004 PMSA International Conference ‘Global Knowledge for Project Management Professionals’*, 183–192.
	[66] Sauer, C., Gemino, A., & Reich, B. H. (2007). The impact of size and volatility on IT project performance. *Communications of the ACM*, *50*(11), 79–84.
	[67] The Standish Group. (2007). Standish Group Report: There's Less Development Chaos Today. *The Standish Group*.
	[68] Emam, K. el, & Koru, G. A. (2008). A replicated survey of IT software project failures. *IEEE Software*, *25*(5), 84–90.
	[69] Varajão, J., Cardoso, J., Gonçalves, D., & Cruz, J. B. (2008). Análise à gestão de projetos de desenvolviemnto de software em grandes empresas portuguesas. *Semana Informática*, *904*, 10–12.
	[70] Maglyas, A., Nikula, U., & Smolander, K. (2010). Comparison of two models of success prediction in software development projects. 6th *Central and Eastern European Software Engineering Conference*, 43–49.
	[48] Alfaadel, F., Alawairdhi, M., & Al-Zyoud, M. (2012). Success and failure of IT projects: a study in Saudi Arabia. 11th WSEAS International Conference on Applied Computer and Applied Computational Science, 77–82.
	[57] Marnewick, C. (2012). A longitudinal analysis of ICT project success. *ACM International Conference Proceeding Series*, 326–334.
	[71] The Standish Group. (2013). CHAOS Manifesto 2013. *The Standish Group*.
	[72] Varajão, J., Dominguez, C., Ribeiro, P. M. G. de A., & Paiva, A. (2014). Failures in software project management – are we alone? A comparison with construction industry. *The Journal of Modern Project Management*, *2*(1), 23–25.
	[62] The Standish Group. (2015). Chaos Report 2015. *The Standish Group*.
	[73] Donkersloot, M., & Gaikema, M. (2018). An international research on IT-projects, to leverage success of Dutch IT-projects. *Informagency*, 1–4.
	[74] Frączkowski, K., Gładysz, B., Kuchta, D., & Stanek, S. (2018). Selection of IT projects to be implemented in an organisation to maximise their success probability. *Journal of Decision Systems*, *27*(1), 111–122.
	[75] The Standish Group. (2018). Decision latency theory: it is all about the interval. *The Standish Group*.
	[77] Laranjeira, M., Trigo, A., & Varajão, J. (2019). Success of Software Development Projects in Portugal - preliminary results. *19.*^*a*^*Conferência Da Associação Portuguesa de Sistemas de Informação (CAPSI’2019)*.
	[78] Khoza, L., & Marnewick, C. (2020). Waterfall and Agile information system project success rates - A South African perspective. *South African Computer Journal*, *32*(1).
	[79] Varajão, J., Trigo, A., Moura, I., & Pereira, J. L. (2020). IS Project Management Success in Developing Countries. *European, Mediterranean, and Middle Eastern Conference on Information Systems*, *402*, 769–780.
	[80] Bilir, C., & Yafez, E. (2021). Project success/failure rates in Turkey. *International Journal of Information Systems and Project Management*, *9*(4), 24–40.
	[29] Varajão, J., Pereira, J. L., Trigo, A., & Moura, I. (2021). Information systems project management success. *International Journal of Information Systems and Project Management*, *9*(4), 62–74.
	[5] Pereira, J., Varajão, J., & Takagi, N. (2022). Evaluation of Information Systems Project Success – Insights from Practitioners. *Information Systems Management*, *39*(2), 138–155.
Other	[41] Pretorius, S., Steyn, H., & Jordaan, J. C. (2012). Project Management Maturity and Project Management Success in the Engineering and Construction Industries in Southern Africa. *South African Journal of Industrial Engineering*, *23*(3), 1–12.
	[72] Varajão, J., Dominguez, C., Ribeiro, P. M. G. de A., & Paiva, A. (2014). Failures in software project management – are we alone? A comparison with construction industry. *The Journal of Modern Project Management*, *2*(1), 23–25.
	[79] Varajão, J., Trigo, A., Moura, I., & Pereira, J. L. (2020). IS Project Management Success in Developing Countries. *European, Mediterranean, and Middle Eastern Conference on Information Systems*, *402*, 769–780.
	[80] Bilir, C., & Yafez, E. (2021). Project success/failure rates in Turkey. *International Journal of Information Systems and Project Management*, *9*(4), 24–40.
Unspecified	[8] Association of Project Management. (2015). Conditions for Project Success: APM research report. *APM Conference*.
	[7] Serrador, P., & Turner, R. (2015). The Relationship Between Project Success and Project Efficiency. *Project Management Journal*, *46*(1), 30–39.
	[76] Hoxha, L., & Mcmahan, C. (2019). The Influence of Project Manager's Age on Project Success. *Journal of Engineering, Project, and Production Management*, *9*(1), 12–19.
	[81] Pretorius, S., Bond-Barnard, T. J., Steyn, H., & Jordaan, J. (2022). Project Success and Project Manager Competency. *Portland International Conference on Management of Engineering and Technology (PICMET)*.
